# Conversion of quinoa and lupin agro-residues into biochar in the Andes: An experimental study in a pilot-scale auger-type reactor

**DOI:** 10.3389/fbioe.2022.1087933

**Published:** 2022-12-05

**Authors:** Mario A. Heredia Salgado, Jonathan A. Coba S, A. Cianferoni, Ina Säumel, Luís A. C. Tarelho

**Affiliations:** ^1^ Integrative Research Institute for Transformation of Human-Environment Systems (IRITHEsys), Humboldt Universität zu Berlin, Berlin, Germany; ^2^ Department of Environment and Planning, Centre for Environmental and Marine Studies (CESAM), University of Aveiro, Aveiro, Portugal; ^3^ Bioenergía de los Andes (BDA), José L. Tamayo y R. Teran. Quito, Ecuador; ^4^ European Comitee for Training and Agriculture (CEFA), Eloy Alfaro y Amazonas, Quito, Ecuador

**Keywords:** pyrolysis, agro residues, biochar, *Chenopodium* quinoa Wild, Lupinus mutabilis Sweet

## Abstract

In the last decades, the cultivation of quinoa and lupin became an important source of income for Andean farmers due to the demand for high nutrient-density foods from the Global North. The increase in the cultivation intensity caused by this exogenous demand led to the overexploitation of local ecosystems and a decrease in soil fertility. As an alternative to recover and improve soil quality, this work uses a pilot-scale auger pyrolysis reactor, implemented in the Andes, to assess the conversion of the agro residues generated in the post-harvesting processes of quinoa and lupin into biochar for soil amendment. Following the European Biochar Certificate guidelines, the pyrolyzed quinoa stems can be classified as biochar while the pyrolyzed quinoa husks can be classified as pyrogenic carbonaceous material. Both can be used for soil amendment considering their molar ratios (H/C_org_, O/C_org_) and carbon content. It was not possible to carbonize lupin stems and seedcases. Despite the altitude (2,632 m.a.s.l), the CO concentration during the carbonization of quinoa stems and husks were 1,024.4 and 559 mg/Nm^3^, this last, near the European eco-design standard of 500 mg/Nm^3^. A subsequent SWOT analysis showed the need to explore low-cost and low-complexity pyrolysis reactors that allow the decentralized conversion of agro residues at the farm-scale. The development of local standards to regulate the production and use of biochar is also essential to grant the safety of the processes, the quality of the products, and mobilize funds that allow implementation at relevant scales.

## 1 Introduction

Quinoa (*Chenopodium quinoa Wild*) and lupin (*Lupinus mutabilis Sweet*) are Andean grains typically cultivated in the highlands of Ecuador, Bolivia, and Peru. Both contain large amounts of protein, dietary fibers, essential fatty acids, vitamins, minerals, and carbohydrates. These Andean grains have been of major importance for the food security of farmer communities living in the Andes where access to meat protein sources is limited (FAO and CIRAD, 2015). Before 2000, the consumption of quinoa and lupin was not usual outside South America (Graf et al., 2016; Kouris-Blazos and Belski, 2016). However, the outstanding quality of their protein -it contains lysine and leucine - besides being gluten-free, positioned these Andean grains as an upper-class food in wealthy countries of the Global North. In these countries the interest in foods with potential health benefits and high nutrient density often referred as “superfoods”, is increasing (Bellemare et al., 2018; Bryant et al., 2022).

Besides the health and nutritional interest, the “superfoods” like quinoa and lupin are also considered alternatives to implement a plant-based diet in the Global North. It is claimed that quinoa consumption can reduce meat production along with the environmental consequences linked with animal farming, for instance, land or forest clearing and enteric methane emissions (Abbis et al., 2017; Stubbs et al., 2018). From 2010, these perceptions concerning Andean grains gave rise a sustained demand from wealthy countries triggering a historical price increase (Bedoya-Perales et al., 2018; Bonifacio et al., 2022). Accordingly, the area dedicated to its cultivation in the producer countries in the Andean highlands increased becoming a relevant source of income for farmers that saw in these crops an opportunity for poverty alleviation (Stensrud, 2019).

Recently, the increasing supply of quinoa and lupin grew in Europe, Africa, and the United States of America has displaced Andean production and the prices have lowered (Jacobsen, 2017; Maliro et al., 2021). Nonetheless, the environmental impacts in the producer regions of the Andes highlands caused by the once commercial success of quinoa and lupin remain, namely, soil over-exploitation, intensive use of fertilizers, and biodiversity loss due to the conversion of typical highland eco-systems into cropland (Jacobsen, 2011; Fuentes et al., 2012; Bellemare et al., 2018). In the Andean highlands, soil overexploitation during the quinoa and lupin boom has also caused a decline in its usually high nutritional density, putting at risk the food security of farmers’ communities (Silva et al., 2020; Bonifacio et al., 2022). Accordingly, the identification of alternatives to improve soil quality and prevent erosion is relevant for the restoration of highland ecosystems and to grant access to quality protein sources for local communities.

It is stated that the agro-residues generated during the post-harvesting processes of quinoa and lupin could be a potential feedstock to produce organic soil amendments such as biochar which is a solid carbonaceous material produced through pyrolysis. There are 2.4 and 7 tons of agro-residues being generated per ton of quinoa and lupin grain threshed, respectively (Heredia et al., 2017). The use of biochar made from these types of agro residues can be an alternative to bring back to the soil part of the carbon and minerals absorbed by the biomass during growth (Scholz et al., 2014). Concerning the use of biochar in quinoa crops, it is reported an increase in biomass yield after application, improvement of soil fertility, reduction of inputs of N-fertilizer, and increase of organic carbon content in the soil (Kammann et al., 2015). The application of biochar on sandy soils, such as the ones used for quinoa and lupin cultivation, promotes the growth of leaf areas, increases drought tolerance and water use efficiency (Kammann et al., 2011; Egamberdieva et al., 2017).

It is worth to note that the studies that refer to the use of biochar as a soil amendment in quinoa and lupin crops, rather than considering locally available residual biomass, that is, the agro-residues generated in the post-harvesting processes, consider feedstocks for the production of biochar such as peanut hull residues (Kammann et al., 2011), residual forest biomass and maize (Egamberdieva et al., 2017), and wood chips made of 80–20 wt% coniferous and deciduous wood (Kammann et al., 2015). These experimental studies on the use of biochar as a soil amendment in quinoa and lupin crops do not consider locally available feedstocks to produce biochar probably because they were performed by foreign research institutions in laboratories not located in the Andes highlands.

In the Andean region, few studies refer to the use of agro-residues generated during the post-harvesting processes of quinoa and lupin as feedstock for biochar production. For instance, a study made in Ecuador through a numerical model (Heredia et al., 2017) shows that the theoretical yield of biochar produced from quinoa and lupin agro-residues ranges from 22.4 wt% to 28.4 wt% when the pyrolysis temperatures are 450°C and 550 C, respectively. This study further shows that the thermal energy required to drive the pyrolysis process can be supplied through the combustion of pyrolysis gases. The referential proximal and elemental composition of the quinoa and lupin agro-residues is shown in Table 1.

**TABLE 1:** Proximate and elemental composition of the agro-residues generated after the post-harvesting processes of quinoa and lupin in the highlands of Ecuador (Heredia et al., 2017).

	Quinoa		Lupin	
	Stem	Husk	Stem	Seedcase
**Proximate Analysis (%wt,**_**wb**_)				
Moisture	2.6	4.8	3.6	4.8
Volatile	77.9	71.9	79.4	72
Ash	5	15.6	4.9	15.6
Fixed carbon^a^	14.5	7.7	12.1	5.9
**Elemental Analysis** (%wt,_db_)				
Ash	5.3	18.5	5.2	18.5
C	46.3	42.6	47	45.7
H	5.7	5.2	5.9	5.8
N	10.7	15.4	11.5	9.7
S	0.5	0.5	0.5	0.4
O^a^	31.5	17.8	29.8	19.9
**Lower Heating Value - LHV** (MJ/kg_db_)	17.8	15.3	17.1	17.5

^a^ Calculated by difference.

Regarding laboratory tests performed in the Andes highlands, there is an experimental study that explored the use of quinoa stems not to obtain biochar, but as feedstock to produce a pelletized solid fuel to be used in rural stoves for food cooking (Alarcon et al., 2017). That study focuses on the mechanical properties of the produced pellets rather than the performance and behavior of the quinoa agro-residue during its thermochemical conversion process. For the case of lupin stems and seedcases, outside the quantification of the rate of agro-residues produced in the post-harvesting process and the characterization of their proximal and elemental composition shown in Table 1, there are no experimental data concerning their use as feedstock for biochar production neither as solid fuel.

Concerning the thermochemical conversion of quinoa stems and husks, there is a thermogravimetric analysis performed by (Paniagua Bermejo et al., 2020) that revealed two stages of weight loss under an oxidative atmosphere. Furthermore, two heat release stages were identified when the experiment was performed under an inert atmosphere. That study claims that the stages of thermochemical conversion were influenced by the content of cellulose and lignin. Following the results of the thermogravimetric analysis performed under oxidative and under an inert atmosphere, the authors state that stems should be preferred as feedstock whether for combustion or carbonization processes.

As shown, to produce biochar from quinoa and lupin agro residues, the reviewed experimental studies use lab-scale reactors, usually fixed beds, which are batch operated and fed with samples of a few grams of biomass (Alarcon et al., 2017; Heredia et al., 2017; Paniagua Bermejo et al., 2020). In these types of bench-scale laboratory research infrastructures, the potential constraints linked with process scale up to practical size and the effect of local conditions, as the altitude, over the process operating conditions can hardly be explored and has not been yet done. A step forward from these numerical and lab-scale studies requires the demonstration of the pyrolysis process at a relevant scale that could provide insights into the feasibility of implementing this technology for the benefit of farmers’ communities of the Andes highlands and the local ecosystems.

In this context, the present study provides new information on using the agro residues generated during the post-harvesting processes of quinoa and lupin, namely, quinoa stems and husks together with lupin stems and husks as feedstocks in a pilot-scale auger pyrolysis reactor implemented in the Andes highlands (capacity 30 kg/h) which was designed for the combined production of biochar and thermal energy. This reactor has been previously demonstrated effective for the conversion of agro-residues produced in palm oil mills, into biochar (Heredia Salgado et al., 2020). The pyrolysis experiments were performed for each feedstock under conditions of relevance for the producer countries in the Andes, namely at 2,632 m.a.s.l. The operating conditions of the pyrolysis process along with the properties of the carbonized products are reported and discussed concerning its use for soil amendment. Flue gas emissions from the pilot-scale reactor are also monitored and assessed according to widely recognized eco-design standards. Finally, a SWOT analysis based on participant observations and expert meetings is included to discuss the extent to which the technology used to produce biochar is adaptable to the context where the quinoa and lupin agro-residues are being generated.

## 2 Materials and methods

### 2.1 Study site and collection of agro-residues

The agro-residues used in the pyrolysis experiments were collected following the participant observation method. The principal author of this work (Mario Heredia) got invited by the community San Francisco de Bishud, province of Chimborazo in Ecuador (S2°17′8.2″ W78°45′30.8”) to participate in the harvest and post-harvesting process of quinoa and lupin. The post-harvesting processes followed the typical tasks performed by farmers in the Andes highlands as described by (Silva et al., 2020) and (FAO and CIRAD, 2015). Thus, after harvesting, the collected quinoa and lupin plants were dried by the stacking method which involves arranging the plants in stacks (cone-shaped mounds). Then, the dried plants were fed to a threshing machine to separate the grain from the panicle.

The collected samples were threshed by the mechanical method using a machine with a capacity of 500 kg/h driven by a gasoline engine. The threshing machine separated the grain from the stems and the husks/seedcases in one single process. The four types of agro-residues collected from the thresher, namely quinoa stems and husk and lupin stems and seedcases, were saved in jute sacks considering batches of 350 kg following the norm UNE-CEN/TS 14778-1:EX.

**FIGURE 1:**
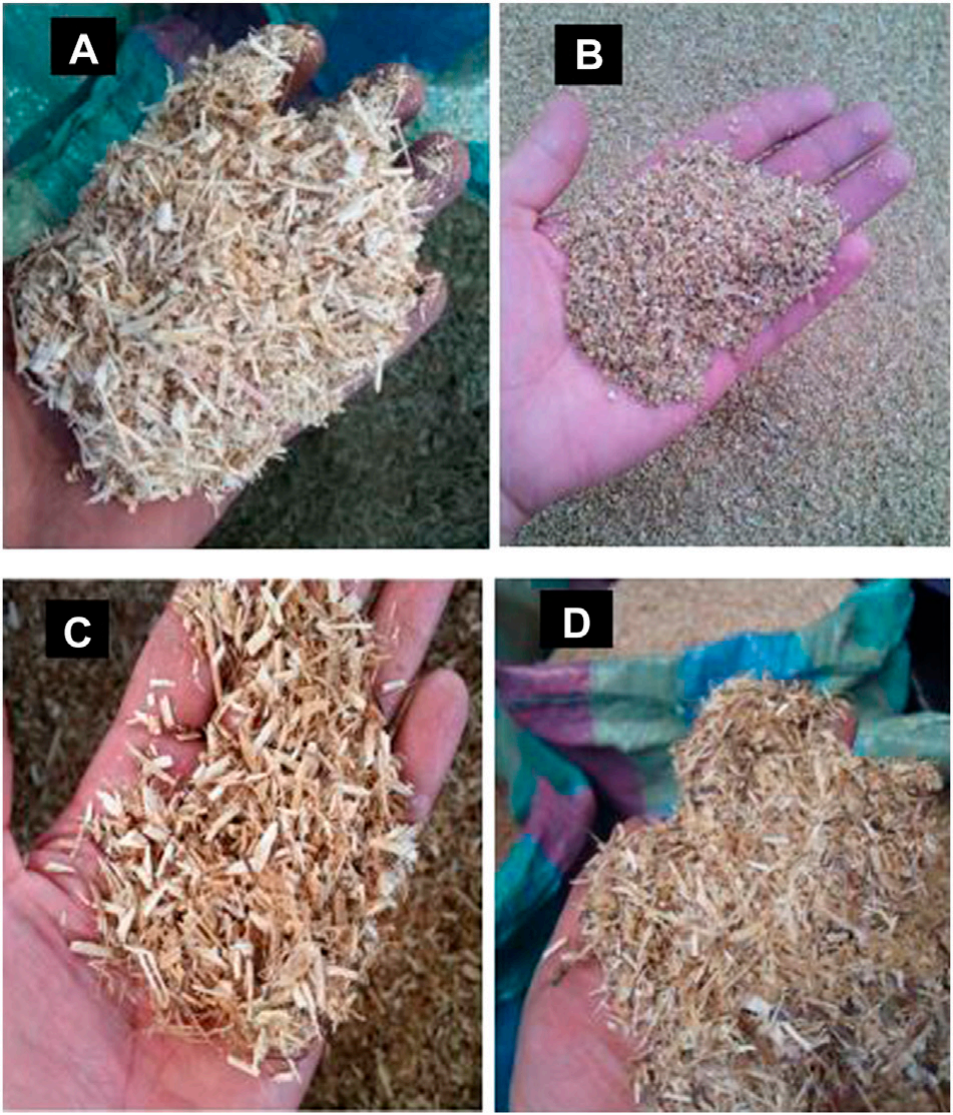
**(A)** Quinoa stems crushed in the hammer mill. **(B)** Quinoa husks collected from the thresher. **(C)** Lupin stems crushed in a hammer mill. **(D)** lupin seedcases collected from th thresher.

After the threshing process, the quinoa and lupin stems resemble sticks with a length between 0.8 and 1.5 m. Before the pyrolysis experiments, these quinoa and lupin stems were crushed in a hammer mill, to a particle size between 5 and 15 mm. The quinoa husks and the lupin seedcases were used in the pyrolysis experiments as collected from the thresher because their particle size was already between 1 and 5 mm. Figure 1 shows the feedstocks used in the pyrolysis experiments, namely quinoa stems (QS), quinoa husks (QH), lupin stems (LS), and lupin seedcases (LSC).

### 2.2 Pilot-scale auger-type pyrolysis reactor

**FIGURE 2:**
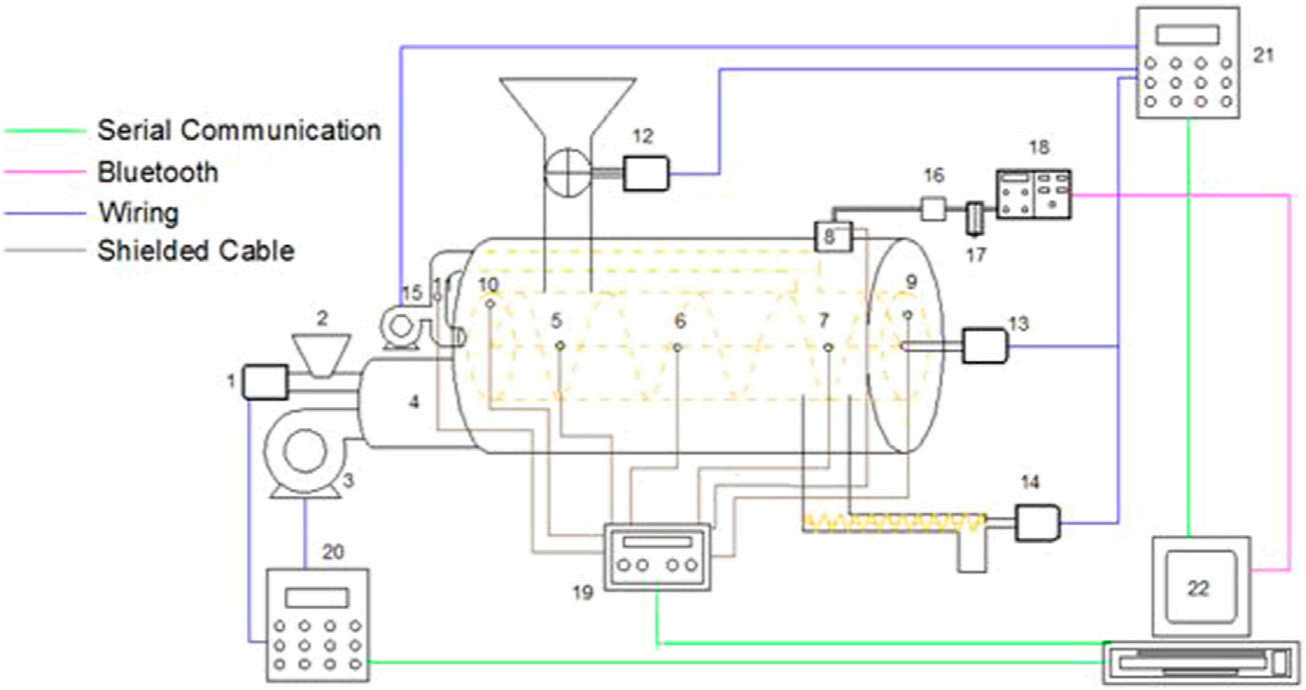
Pilot-scale auger-type pyrolysis reactor used to produce biochar from agro-residues generated during the post-harvesting processes of quinoa and lupin. Legend: 1. HBP feeder, 2. HBP hopper, 3. HBP blower, 4. HBP frame, 5. Combustion chamber thermocouple (T1), 6. Combustion chamber thermocouple (T2), 7. Combustion chamber thermocouple (T3), 8. Chimney thermocouple (T4), 9. Carbonization chamber outlet thermocouple (T5), 10. Carbonization chamber inlet thermocouple (T6), 11. Pyrolysis gas thermocouple (17), 12. Rotary vane valve, 13. Auger shaft and motor, 14. Discharge valve, 15 Pyrolysis gas burner, 16. Particle filter, 17. Condenser filter. 18. On line gas analyzer 19. Thermocouples data logger, 20. Combustion module controller, 21. Pyrolysis module controller, 22. Computer. Adapted from (Heredia Salgado et al., 2020).

The agro-residues described in Section 2.1 were pyrolyzed in a pilot-scale auger-type reactor previously used to convert agro residues generated in palm oil mills into biochar (Heredia Salgado et al., 2020). This research facility located at 2,632 m.a.s.l (S 0°17′30.8´´ W78°30′7.9´´) which are relevant conditions to assess potential constraints in the implementation of pyrolysis processes in the Andes highlands. Further specifications, heat exchange methods and constructive details of devices, systems and sub systems of the pilot-scale auger-type pyrolysis reactor can be consulted elsewhere (Heredia Salgado, 2020). The pilot-scale auger-type pyrolysis reactor used in the pyrolysis experiments is composed of two integrated modules of thermochemical conversion, namely, a combustion module and a pyrolysis module. The combustion module consists of a horizontal burner prototype (HBP) (numbers one to four in Figure 2) that uses a fraction of the agro-residues as a solid fuel to produce the thermal energy required to heat the pyrolysis module and start the pyrolysis process. Details about the HBP can be found in previous works, namely (Heredia Salgado et al., 2019a) and (Heredia Salgado et al., 2019b).

The pyrolysis module includes the ancillaries required to feed the agro-residues (number 12 in Figure 2), discharge the biochar (number 14 in Figure 2), and those required for the energetic conversion of pyrolysis gas by combustion, that is, a pyrolysis gas burner (PGB) (number 15 in Figure 2). Accordingly, the HBP and the PGB share the combustion chamber where the hot gases generated by the combustion processes -whether from solid agro-residues or pyrolysis gases-supply the heat required by the pyrolysis process. The flue gas generated during these combustion processes is discharged into the atmosphere through a chimney (number eight in Figure 2).

The residual biomass used for the experiments is fed to the pyrolysis module from a hopper using a rotary vane valve (number 12 in Figure 2). The biomass feed rate of this valve for all the experiments was 30 kg/h. Additional gases were not used to promote an inert atmosphere in the pyrolysis module nor to drag the pyrolytic gases. The tightness of the pyrolysis module was assured by keeping a constant level of agro-residues in the hopper and owing to the seals placed in the vanes of the rotary valve. Finally, the hopper cover forms a seal of three stages.

After the agro-residues have passed through the rotary vane valve, the auger rotation (number 13 in Figure 2) moves it along the horizontal axis until the end of the pyrolysis chamber. The residence time of the agro-residues within the pyrolysis chamber for all the experiments was 15 min. Then, the carbonized agro-residues (the biochar) are discharged by a coil conveyor (item 13 in Figure 2). A control volume of biochar is always kept over the level of the coil conveyor to avoid air entrance along with a water seal. During the discharge, the water seal is replaced by a nozzle that sprays water over the biochar to prevent dust formation and auto-ignition.

The pilot-scale pyrolysis reactor is operated through two independent controllers namely: a controller for the combustion module and a controller for the pyrolysis module. The controller of the combustion module (number 20 in Figure 2) manages exclusively the ignition process, operating conditions, and thermal output power of the HBP. The controller of the pyrolysis module (number 21 in Figure 2) manages the activation, rotation speed, and rotation direction of the rotary vane valve, the auger, the discharge valve, the air blower of the PGB, and the water nozzle. The changes in the operating parameters implemented in the pyrolysis and combustion controllers are sent through a serial protocol towards a computer interface (number 22 in Figure 2) where they are recorded for later analysis.

### 2.3 Sampling process of pyrolyzed agro-residues

During the pyrolysis experiments, biochar samples of 3.5 kg were collected from the discharge port (number 14 in Figure 2) following the norm UNE-CEN/TS 14778-1:EX. The moisture, ash, and volatile matter content were determined following the standards BS EN 14774-3:2009, BS EN 14775:2009, BS EN 15148:2009, respectively. The heating value and the composition of C-H-N-S of the biochar were determined following the standards ASTM D 1989-96 and BS EN 15104:2011, respectively. The quality of the biochar produced in the experiments as a soil amendment for the quinoa and lupin crops, namely its stability in the soil (molar ratio O/C_org_), carbon content, and its degree of carbonization (molar ratio H/C_org_) were assessed following the European guidelines for the sustainable production of biochar (European Biochar Foundation, 2018). All the samples of biochar were collected during the periods of operation in steady state. Therefore, the biochar was collected when constant temperatures were observed in the combustion and pyrolysis modules along with a steady composition of the flue gas.

### 2.4 Process monitoring: Temperature profiles and flue gas composition

The composition of the flue gas was monitored using an AU Mobile Brain Bee infrared online analyzer (number 18 in Figure 2). The gas analyzer monitors: CO (0–9.99%vol), CO_2_ (0–19.9%vol), HC (0–13,999 ppm, expressed as hexane), and O_2_ (0–24.99%vol). The gas analyzer resolution is 0.01%vol for CO and O_2_, 0.1%vol for CO_2,_ and 1ppm for HC. A particle matter filter followed by a gas condenser submerged in cold water for moisture and condensable material removal was placed before the gas analyzer (see numbers 16 and 17 in Figure 2). The flue gas analyzer communicates with a computer interface (number 22 in Figure 2) where the gas concentrations are plotted in real-time and recorded for later analysis. The conversion efficiency of the combustion processes was assessed by monitoring the CO concentration in the flue gas following the European emissions standards for fixed combustion sources (The European Commission, 2015). Accordingly, the CO concentration in the flue gas was corrected to an O_2_ concentration of 11%vol O_2 dry gas_ (Heredia Salgado et al., 2019a).

Seven thermocouples were distributed between the combustion and pyrolysis modules to monitor the temperature profiles of the combustion and pyrolysis processes. K-type thermocouples were used with a measurement range between 95 and 1,260 C and an accuracy of 2.2°C. Three thermocouples were placed in the horizontal axis of the combustion chamber, namely: thermocouple 1 (number five in Figure 2) is at 0.27 m from the HBP exit, thermocouple 2 (number six in Figure 2) is at 0.55 m from thermocouple 1, and thermocouple 3 (number seven in Figure 2) is at 0.55 m from thermocouple 2. Thermocouple 4 (number eight in Figure 2) was placed in the exit flue gas duct to further calculate an estimative of thermal energy associated with the exiting combustion flue gases. The thermocouples used to monitor the temperature in the combustion module have a length of 100 mm and 5 mm in thickness.

The temperatures in the pyrolysis module were monitored at the inlet and outlet of the auger within the pyrolysis chamber and were placed through the front and rear covers of the combustion chamber (see numbers 9 and 10 in Figure 2). The thermocouple located in the front cover of the combustion chamber, that is at the HBP side, is 300 mm in length, 5 mm in thickness, and is located at 140 mm up from the central axis of the auger shaft. The thermocouple located in the rear cover of the combustion chamber is 100 mm in length, 5 mm in thickness, and is located 140 mm up from the auger shaft. The temperature of the pyrolysis gas was monitored at the suction duct of the PGB with a thermocouple of 25 mm length and 5 mm thickness (number 11 in Figure 2). The temperature signal of the seven thermocouples is acquired with an interval of one second and sent through a temperature datalogger (number 19 in Figure 2) by serial communication to the computer interface (number 22 in Figure 2) to be plotted in real-time and recorded for later analysis.

### 2.5 SWOT analysis: Internal capabilities and external constraints linked with the local communities concerning biochar production

The reviewed studies about the use of biochar in quinoa and lupin crops barely consider the local knowledge and ignore the available residual biomass (see Section 1). In this sense, this SWOT analysis is intended to grasp whether biochar can be effectively implemented in the Andes highlands as an alternative for soil amendment. To do so, it relies on the participant observations made by the principal author (Mario Heredia) who participated in a complete cycle of quinoa and lupin harvesting and post-harvesting process, invited by an Andean community (see Section 2.1).

Accordingly, information on the execution of harvesting and post-harvesting processes in the community along with the procedures and machinery involved were registered with photographs and field notes. Data concerning the current uses of the agro residues generated during the threshing process and soil fertilization practices were gathered using open interviews with the farmers involved in the threshing processes. The SWOT analysis was then complemented with the criteria of practitioners and experts from international cooperation agencies with experience in the cooperative sector linked with quinoa and lupin, specifically from the European Committee for Training and Agriculture (CEFA) in Ecuador. References from the technical literature published in Spanish by local research institutions, namely the Instituto Nacional de Investigaciones Agropecuarias (INIAP) in Ecuador were also included in the SWOT analysis as contrast and verification of the information gathered through the participant observation method (Caicedo and Peralta, 2000; Caicedo et al., 2001; Peralta et al., 2012).

## 3 Results and discussion

### 3.1 Pilot-scale auger-type pyrolysis reactor: Heating process

As shown in Section 2.2, the pilot-scale auger-type pyrolysis reactor has two modules, namely a combustion module and a pyrolysis module. In the combustion module, the reactor uses a fraction of the agro residues to produce the thermal energy required to heat the pyrolysis module to the point that the carbonization process is maintained under steady conditions. Therefore, during the first stage of the experimental work, four independent experiments were considered in the combustion module using QS, QH, LS, and LSC as solid fuels. Once the pyrolysis module is hot, the feed of QS, QH, LS, and LSC towards the pyrolysis chamber was tested individually. Table 2 shows the observations made while conveying these agro-residues from the hopper of the combustion module towards the combustion bed and from the hopper of the pyrolysis module towards the pyrolysis chamber.

**TABLE 2:** Observations made during the conveying process of QH, QS, LS and LSC from the hopper of the combustion module towards the combustion bed and from the pyrolysis module hopper towards the carbonization chamber.

Feedstock	Feeding	Combustion Module: Observation	Feeding	Pyrolysis module: Observation
Quinoa stems (QS)	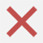	Dragging: QS particles reach the combustion bed. Afterward, QS was dragged by the combustion air stream out of the combustion chamber	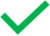	QS was fed at a steady rate of 30 kg/h
Quinoa husks (QH)	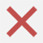	Dragging: QS particles reach the combustion bed. Then, QS was dragged by the combustion air stream out of the combustion chamber	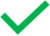	QH was fed at a steady rate of 30 kg/h
Lupin stems (LS)	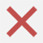	Bridging: irregular flow of particles in the hopper, stagnant regions, and dome formation	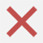	Bridging: irregular flow of particles in the hopper, stagnantregions, and dome formation. Leakage of pyrolysis gas through the hopper cover. Vapor condensation within the hopper moist the feedstock
Lupin seedcases (LSC)	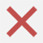	Bridging: irregular flow of particles in the hopper, stagnant regions, and dome formation	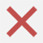	Bridging: irregular flow of particles in the hopper, stagnant regions, and dome formation. Leakage of pyrolysis gas through the hopper cover. Vapor condensation within the hopper moist the feedstock

Regarding the combustion module, it was observed that LS and LSC are difficult to transport from the HBP hopper toward the combustion bed. Irregular flow in the hopper was observed due to stagnant regions of particles that tend to adhere to the hopper walls regardless of the surface angle implemented. These stagnate regions of LS and LSC within the hopper resulted in the reported “bridging” or “dome” formation (Dai et al., 2012) which causes an intermittent and inconsistent feed towards the combustion bed.

Unlike the lupin agro-residues, QS and QH were constantly conveyed from the hopper of the combustion module towards the HBP bed. Nonetheless, the air stream provided by the blower linked with the HBP during the low-temperature ignition process dragged most of the QS and QH particles, that already reached the combustion bed, out of the burner. The issues concerning particles dragging by the stream of combustion air are typical of biomass-derived fuels with low particle density as QS and QH (Polonini et al., 2019).

This dragging and dome effects linked with QS, QH, LS, and LSC caused problems to ignite these agro residues in the combustion module and it was not possible to fixate a steady flame front. Accordingly, QS, QH, LS, and LSC were discarded as fuel sources for the initial heating process of the pilot-scale auger-type pyrolysis reactor. The reactor hoppers do not have stirring mechanisms, and thus the flow of agro-residues particles in the hopper of the combustion module depends mainly on gravity. Accordingly, an agro-residue with higher bulk density had to be used as solid fuel in the combustion module during the initial heating process of the pilot-scale auger-type pyrolysis reactor, namely palm oil kernel shell. This decision follows the initial demonstration of the operation of this pilot-scale auger-type reactor in which the initial heating process of the pyrolysis chamber was performed by feeding palm oil kernel shells (1,120 kg/m^3^) in the combustion module (Heredia Salgado et al., 2020).

Thus, the heating process increase the temperature of the combustion chamber to 550 C (thermocouple T3 in Figure 2). The corresponding temperature at the inlet of the carbonization chamber was 400 C (thermocouple T6 in Figure 2). Under these conditions, the rotary vane valve of the pyrolysis module (number 12 in Figure 2) was activated to start conveying QS, QH, LS, and LSC towards the pyrolysis chamber in individual experiments.

### 3.2 Pilot-scale auger-type pyrolysis reactor: The pyrolysis module

During the pyrolysis experiments using LS and LSC as feedstock, a constant void in the center of the biomass hopper of the pyrolysis module was observed (see Table 2). As the feed of the LS and LSC towards the pyrolysis module starts, a mass of static material develops around a void in the center of the hopper through which the lupin agro residues eventually flow, that is, the often-mentioned bridging, arching, or rathole effect (Dai et al., 2012).

As described in Section 2.2. The pilot-scale auger-type reactor does not use additional gases to secure an inert atmosphere in the pyrolysis module because the feedstock in the secondary hopper, the rotary vane valve, and a sealed cover in the hopper act as a triple seal that grants tightness. The voids observed in the hopper while conveying LS and LSC causes the pyrolysis gas to bypass the rotary vane valve and slight pyrolysis gas leakages were observed through the hopper cover in the pyrolysis module. Fifteen minutes after starting to convey the LS and LSC, the condensable species in the pyrolysis gas (i.e., water and tar), starts condensing in the hopper cover of the pyrolysis module and falling over the agro-residues (LS and LSC) within the hopper. This condensing effect moistens the agro-residues in the hopper, turning impossible to feed them through the rotary vane valve towards the pyrolysis chamber. Furthermore, the high temperature of the pyrolysis gases accumulated in the hopper caused the failure of the hopper cover seals, and significant pyrolysis gas leakages from the hopper were observed thereafter.

The unsteady supply of LS and LSC towards the pyrolysis chamber and the leakages of pyrolysis gas from the hopper cover caused an unsteady carbonization process. Furthermore, the temperatures within the combustion and pyrolysis modules did not reach the values required to achieve an auto-thermal operation mode, as reported by Heredia Salgado, 2020, during the operation of the same reactor with agro-residues of high particle density. Therefore, the carbonization experiments with LS and LSC were suspended.

**FIGURE 3:**
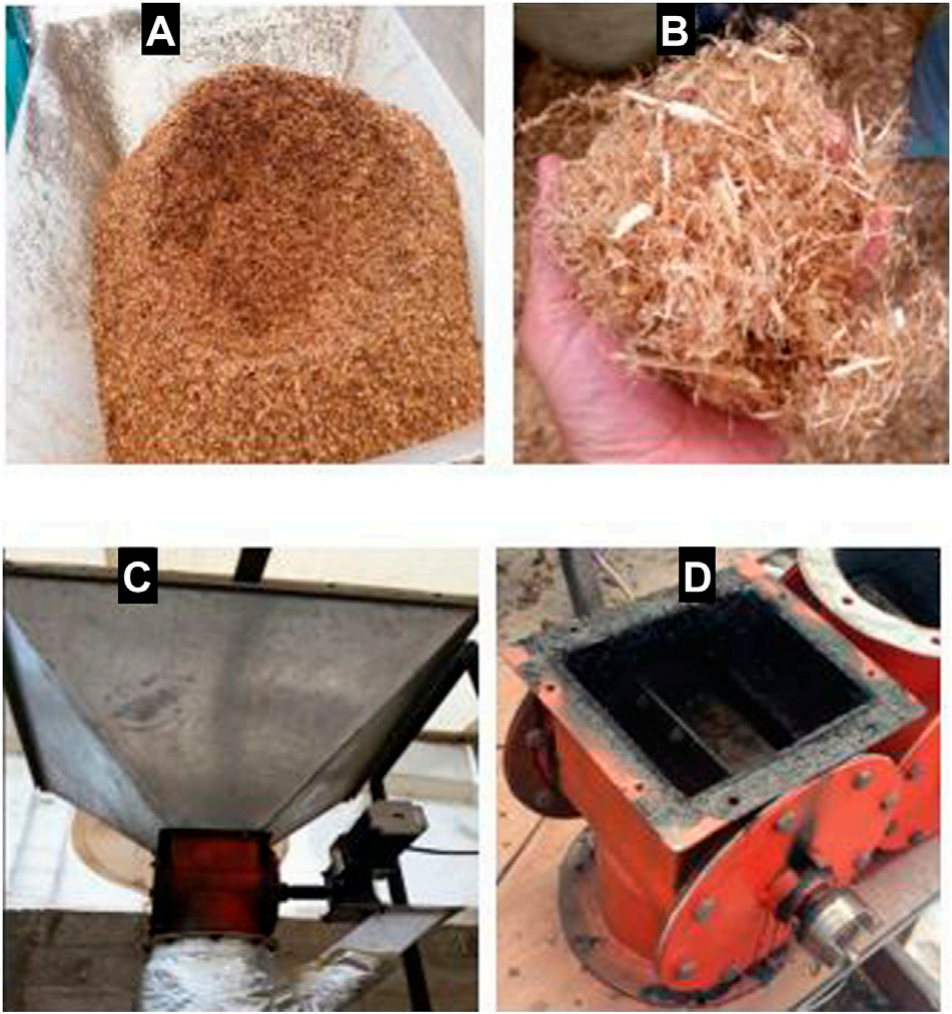
**(A)** Mass of static bulk material around a void observed in the center in the pyrolysis module hopper while conveying LS and LSC towards the carbonization chamber. **(B)** Scourers found in the hopper that result from the interaction between LS and LSC particles with small broken fibers. **(C)** Pyrolysis module hopper with cover and rotary valve. **(D)** Failure of seals and tars condensed in the rotary vane valve.

It is worth mentioning that during the grinding of LS, elongated fibers were detected within the mill. These long fibbers became continuously entangled in the rotor of the mill making it difficult to reduce and adjust the granulometry to the desired particle size. Although these long fibers of the LS were manually removed during the grinding process, a remaining fraction of broken fibers of smaller size eventually passed the outlet mesh of the mill. During the pyrolysis experiments with LS, the movement of material in the hopper revealed that these remaining fractions of broken fibers interact with the larger LS particles and tend to form small agglomerates like scourers (see Figure 3). The trend to form these scourers in the hopper is the main cause of the transporting problems, that is, the dynamic bridging that prevented the use of LS as feedstock to produce biochar in the pilot-scale auger-type pyrolysis reactor. This dynamic bridging effect was also observed in experiments that used LSC as feedstock.

### 3.3 Pyrolysis of quinoa stems and husks

As shown in Table 2, QS and QH were fed from the pyrolysis module hopper towards the carbonization chamber under steady-state conditions and without a bridging effect. The QS and QH were fed when the temperature at the inlet of the pyrolysis chamber was 400 C (thermocouple T6 in Figure 2). During the experiments, the mass flow of QS and QH was 30 kg/h and the residence time of the feedstock within the pyrolysis chamber was 15 min. As the feedstock is distributed along the pyrolysis chamber, the gases generated during the initial steps of devolatilization are dragged by the PGB (number 15 in Figure 2), from the pyrolysis chamber towards the combustion chamber. Accordingly, the thermal energy required by the pyrolysis process is supplied by the hot gases generated from the combustion of pyrolysis gases and the combustion of biomass fuel in the HBP, that is, a co-combustion condition.

During the pyrolysis of palm oil kernel shells, it is possible to shift from this co-combustion stage to an auto-thermal condition in which the thermal energy required by the pyrolysis process is supplied exclusively by the combustion of the pyrolysis gases (Heredia Salgado et al., 2020). To shift from the co-combustion stage towards the auto-thermal operation mode, the thermal power output of the HBP is decreased progressively as the temperature in the combustion chamber increases due to the increase in the yield of pyrolysis gas. Nonetheless, during the co-combustion stage corresponding to the experiments with QS and QH, although there is a steady flame front in the PGB, the progressive decrease of the PGB thermal power output caused a decrease in the temperatures of the combustion (thermocouples T1, T2 and T3 in Figure 2) and pyrolysis chambers (thermocouples T5, T6 and T7 in Figure 2). Consequently, the pyrolysis process was not maintained under steady-state conditions and the flame front of the PGB was extinguished 10 min after turning off the HBP.

During the experiments that use QS and QH, the HBP remained on to maintain steady temperatures in the combustion and pyrolysis chambers. The feeding rate of solid fuel (palm kernel shell) implemented in the HBP to support the combustion process in the PGB was 4.5 kg/h. Under these operating conditions, a steady flame front in the PGB was observed and the pilot-scale auger-type pyrolysis reactor reaches steady-state operation. The mean temperatures observed in the combustion and pyrolysis modules during the experiments with QS, QH and the corresponding standard deviation is shown in Table 3. It is important to note that thermocouples T5 and T6 shown in Table 3 are meant to represent the temperature in this zone of the reactor which is not necessarily the temperature of the feedstock particles flowing through the pyrolysis chamber. The technical limitation that implies the installation of a thermocouple in direct contact with the particles moving along the pyrolysis chamber, and whether this measurement is representative of the temperature of the particles in different positions within the reactor, is a limitation typically associated with continuous and semicontinuous pilot-scale pyrolysis reactors as the one used in this study (Brassard et al., 2017; Campuzano et al., 2019). As an alternative to overcome this limitation, the use of computational fluid dynamics may aid the exploration of the temperature distribution of the particles within the pyrolysis chamber (Aramideh et al., 2015).

**TABLE 3:** Temperatures (mean and standard deviation) observed during the pyrolysis process of QS, and QH in the pilot-scale auger-type reactor. Location of thermocouples in the reactor is shown in Fig.2.

Temperature (°C)	Quinoa stems (QS)		Quinoa husks (QH)	
	Mean	Standard deviation	Mean	Standard deviation
**Combustion chamber, T1**	418.4	22.4	419.6	26.2
**Combustion chamber, T2**	368.6	15	389.6	15.9
**Combustion chamber, T3**	554.3	41.5	563.5	47.9
**Flue gas, T4**	402.5	50.8	435.1	54.1
**Pyrolysis chamber outlet, T5**	500.4	7.7	500	37.6
**Pyrolysis chamber inlet, T6**	435.3	36.8	426.2	42.7
**Pyrolysis gas, T7**	235.9	21.7	209.3	22.9

In general, the temperatures observed in the combustion and pyrolysis modules during the carbonization of QS, and QH (see thermocouples T5 and T6 in Table 3) are lower than those observed during the carbonization process of palm oil kernel shells, which were up to 600 C in the same reactor type (Heredia Salgado et al., 2020). In the current study, the inability to raise the temperature of the pyrolysis chamber above 500 C and the fact of not reaching the auto-thermal operation condition can be related to the physical and chemical properties of the pyrolysis gas. It should be noted that the physical and chemical composition of the feedstock of the pyrolysis process influences the properties, composition, and LHV of the pyrolysis gas as reported by (Rosas et al., 2014) and (Dunnigan et al., 2018). It is recognized that the lower heating value (LHV) of the pyrolysis gas decreases as the pyrolysis temperature decreases. Furthermore, the content of pyrolytic water in the pyrolysis gas is higher at pyrolysis temperatures below 500 C (Neves et al., 2011). Accordingly, the gas generated during the pyrolysis of QS and QH at a temperature of 500 C is expected to have a lower LHV and a higher content of pyrolytic water than the gas generated during the pyrolysis of palm oil kernel shells at temperatures of 600 C. These changes in the composition of pyrolysis gas that are linked with the pyrolysis temperatures will influence the performance of the combustion module.

The mean concentration of CO, CO_2_, and HC observed during the pyrolysis experiments of QS and QH (co-combustion condition) in a monitoring period of 4 hours under steady-state conditions is shown in Table 4. There was a difference in the concentration of CO in the flue gas for the carbonization experiment that used QH and QS, namely 559 and 1,024.4 mg/Nm^3^ (at 11% vol. O_2_, dry gases), respectively. The particle dynamics of the QS and QH in the hopper of the pyrolysis module influenced this difference between the two sets of experiments shown in Table 4. Although QS and QH were successfully conveyed from the biomass hopper towards the pyrolysis chamber, practically no voids were observed in the biomass hopper during the pyrolysis experiment of QH. The lower tendency of the QH particles to form voids became evident because, unlike QS, the pyrolysis gas that eventually bypass the seals of the pyrolysis module and leaks through the cover of the hopper was not noticeable in the pyrolysis experiment that used QH. This observation is attributed to the better dynamic flowing properties of the QH particles over the QS particles which resulted in a steadier feed toward the pyrolysis chamber. Thus, the better feeding conditions of QH particles can justify the lower CO and HC concentration in the combustion flue gases observed in the corresponding pyrolysis experiment. As observed before in this type of reactor, a steady feed of agro-residues is fundamental to achieve a good conversion efficiency of the pyrolysis gases. Considering a similar feed rate of 30 kg/h in the same type of reactor, the CO concentration in the flue gas observed during the pyrolysis process of palm oil kernel shells was 197 mg/Nm^3^ (at 11% vol. O_2_, dry gases) (Heredia Salgado et al., 2020).

**TABLE 4:** Flue gas composition (mean and standard deviation) observed during the pyrolysis experiments with QS, and QH.

	CO (mg/Nm^3^,_dry gas_, at 11% O_2_, _dry gas_)		HC (mg/Nm^3^,_dry gas_, at 11% O_2_, _dry gas_)		CO_2_ (mg/Nm^3^,_dry gas_, at 11% O_2_, _dry gas_)	
	Mean	Standard deviation	Mean	Standard deviation	Mean	Standard deviation
QS	1,024.4	658.6	35.3	4.1	180,000	2,701.4
QH	559	387.3	20.4	7.1	172,317.1	2,516.7

*The CO, HC, and CO_2_ concentration is presented according to the implementing directive of the European Parliament concerning eco-design requirements for solid fuel boilers (500 mg/Nm^3^), that is, corrected to an O_2_ concentration in the flue gas of 11 %vol, dry gas.

As shown in Table 4, the CO concentration in the combustion flue gas during the experiment of pyrolysis of QS almost doubles the CO concentration in the combustion flue gas observed during the experiment of pyrolysis of QH, and the limit of 500 mg/Nm^3^ referred to in the European eco-design standard was exceeded. It is not clear if the eco-design standard considered (The European Commission, 2015), that is usually applied for boilers and space heaters that use solid fuels and operate at sea level, may be applied for the operating condition implemented in this study, namely co-combustion of gaseous and solid fuels at an altitude of 2,634 m.a.s.l. In this study, the O_2_ concentration in the atmospheric air supplied towards the burners (HBP and PGB) decreases from 21% to 16% due to the decrease of the atmospheric pressure corresponding to an altitude of 2,634 m.a.s.l (Heredia Salgado et al., 2019a). Furthermore, the flue gas concentration shown in Table 4 corresponds to a co-combustion operating condition, that is, the combined operation of the HBP using palm oil kernel shells as fuel and the PGB using the gas generated in the pyrolysis chamber as fuel. Although the limitations of the eco-design standard concerning the context of the study, the exploration of alternatives to increase the combustion temperatures (T1, T2 and T3 in Table 3) and thus, improve the conversion degree of the flammable species as CO and HC is of relevance. For instance, the implementation of air stagging techniques (Qiu, 2013), the alteration of combustion chamber design to increase the residence time of flammable gases and flue gas recirculation (Míguez et al., 2012).

### 3.4 Properties and classification of the solid carbonaceous materials produced in the pyrolysis experiments using QS and QH as feedstock

Table 5 shows the proximal and elemental analysis of the solid carbonaceous materials produced from QS and QH pyrolysis. Usually, a concern regarding the use of the carbonaceous materials produced by pyrolysis in soil applications has to do with the content of volatile organic compounds, that is, tars that condense on their surface (Zheng et al., 2019). The properties of the carbonaceous materials produced by pyrolysis, as the content of volatile organic compounds, is affected mainly by pyrolysis temperature and feedstock type (Tomczyk et al., 2020). In this regard, reach a temperature in the pyrolysis process of at least 400 C is critical to reduce toxicity of the produced carbonaceous materials making them suitable for soil application (Lyu et al., 2016). Table 3 shows that the temperatures at which the carbonaceous materials made from QH and QS were produced, that is, the temperatures at the inlet and outlet of the pyrolysis chamber, were between 426.2 and 500 C, respectively (see T5 and T6 in Table 3). Furthermore, the fluctuation of temperatures at the inlet and outlet of the pyrolysis chamber was never above 8% (see Table 3), being that the guidelines for the sustainable production of biochar allow a fluctuation up to 20%. Accordingly, the volatile matter of the produced carbonaceous materials is seven and three times lower than the volatile matter content of the raw QS and QH, respectively (see Tables 1 and 5). Despite the important reduction of the volatile matter content that result of implementing proper operating temperatures, an estimation of the content of volatile organic compounds by thermal-gravimetric-analysis could also be performed as a further indicator for the evaluation of the pyrolysis process and the quality of the carbonaceous materials obtained.

**TABLE 5:** Proximate and elemental analysis the biochar produced during the pyrolysis experiments of quinoa stems (QS) and the PCM produced during the pyrolysis of quinoa husks (QH).

Proximate analysis (%wt, wet basis)	Quinoa stems (QS) biochar	Quinoa husk (QH) PCM
Moisture	10.2	11
Volatile matter	11.5	20.9
Ash	22.1	30.1
Fixed carbon^a^	56.2	38
**Ultimate Analysis (%wt, dry basis)**		
Ash	28.4	43.1
C	54.9	40.4
H	2.2	3.6
N	1	1.8
S^b^	nd	0.3
O^a^	13.5	10.8
**Lower Heating Value (MJ/kg, dry basis)**	23.7	15
**Biochar molar ratios**		
H/C_org_	0.5	1.1
O/C_org_	0.2	0.2

^a^ Calculated by difference.

^b^ Below the detection limit of the method 100 ppm wt. nd-not determined.

Table 5 shows that the solid carbonaceous material produced from QS have a carbon content higher than 50 wt%, an O/C_org_ ratio lower than 0.4, and an H/C_org_ ratio lower than 0.7. Accordingly, it meets the biochar properties following the European standard (European Biochar Foundation, 2018). Concerning the solid carbonaceous material made from QH pyrolysis, the carbon content is lower than 50 wt% and the H/C_org_ molar ratio (degree of carbonization) is higher than the limit of 0.7 suggested by the European guidelines for the sustainable production of biochar (European Biochar Foundation, 2018). These particular properties can be linked with an incomplete pyrolysis process, namely inadequate residence time and low pyrolysis temperatures. However, temperatures up to 500 C (see Table 3) were registered during the pyrolysis of QH, which agree with the European standards and studies that report positive effects of adding biochar -produced at these conditions-to quinoa and lupin crops (Kammann et al., 2011, 2015; Egamberdieva et al., 2017). In these cases, the European standard used in this study state that the use of mineral-rich materials as feedstock may result in solid carbonaceous materials with high ash and low carbon content and classify them, rather than biochar, as pyrogenic carbonaceous materials (PCMs) (European Biochar Foundation, 2018). This does mean that the carbonized QH can be classified as a PCM and could be used for soil amendment. The PCMs have high nutrient content, therefore representing a valuable product for soil amendment (Paz-Ferreiro et al., 2018). It should be noted that the ash content of the raw QH is higher than that of QS (see Table 1). Consequently, the ash content of the PCM made from QH is around two times higher than that of the biochar made from QS (see Table 4). Therefore, the differences in the carbon content and the H/C_org_ molar ratio observed between the PCM made from QH and the biochar made from QS are not related to an incomplete or inadequate pyrolysis process but rather related to high ash content.

### 3.5 Learning from farmers and local practitioners through the lens of SWOT analysis

In the Andes highlands, before the implementation of a pyrolysis process to convert agro residues into biochar one must consider that unlike traditional commodities such as cocoa, palm oil, or sugar cane, quinoa, and lupin are not large-scale monocultures. Both are cultivated in small-scale farms geographically dispersed along the territory and there are no centralized facilities dedicated to collect and process the panicles, neither for drying nor the threshing process. Accordingly, threshing is a farm delivery service in which the threshing machines are transported from one farm to another using small trucks. After the threshing process, the agro residues accumulate forming small mounts that remain on the many farms of the region to rot, burn in the open or as a low-quality source of organic matter for the soil. Accordingly, the SWOT analysis shown in Table 6 argues that the implementation of a centralized infrastructure for the conversion of agro residues into products for soil amendment as biochar or PCMs would demand collection and transporting operations, which in the case of agro residues with low bulk-density, is costly and inefficient (Chen et al., 2015). Along with the collection and transport operations, the milling processes required to reduce the particle size before pyrolysis in auger-type pyrolysis reactors as the one used in this study (see Section 2.1) also represent a weakness as the initial investment costs and the operating costs will increase.

**TABLE 6:** Results of a SWOT analysis that explore the constraints and prospects concerning the conversion of agro-residues produced during the post-harvesting processes of Quinoa and Lupin into biochar for soil amendment in the Andes highlands.

	Strengths	Weaknesses
**Internal**	- Availability of reasonable quantities of already dry-agro residues - The biochar made from QS and the PCM made from QH fulfill the international requirements to be safely used for soil amendment - Biochar and PCM made from local agro residues can be used to prevent erosion of Andean soils and mitigate, in part, the environmental impacts linked with the past boom of quinoa and lupin crops - Currently, farmers of the highlands produce bio inputs as bokashi, vermicompost, and liquid fertilizers “biol” which can be improved-complemented including biochar and PCMs as an additive - Despite the altitude (2,634 m.a.s.l.), the flue gas during the pyrolysis process follows the European eco-design standards - Ecuador has knowledge concerning high-complexity pyrolysis technologies for biochar production. namely, pilot-scale auger-type reactors. Accordingly, the study, adaptation, and deployment of low-cost and complexity reactors should not be a constraint	- The threshing process is decentralized. There is not a single facility that accumulates and processes the panicles. Thus, agro residues are scattered throughout the territory - An auger-type pyrolysis reactor requires an electricity supply to power electric devices and for automation. Electricity supply in the farms where agro-residues are accumulated is scarce. Thus, there will be constraints in setting up complex reactors in a decentralized operation model in the Andes highlands - The agro residues must be milled to reduce the particle size before pyrolysis. High initial investment costs and operation costs linked with auger-type pyrolysis reactors - The properties and quality of the biochar produced in low-cost and complexity reactors (TLUD´s, flame curtain kilns) may be heterogeneous or not within the guidelines for its safe use in soils - The available agro residues (QH, QS, LS, LSC) could not be used as fuel sources for the initial heating process of the pilot-scale auger-type pyrolysis reactor, demanding the use of alternative solid fuels not necessarily at the reach of farmers in the highlands

	**Opportunities**	**Threats**
**External**	- Biochar made from QS pyrolysis and PCM made from QH pyrolysis are alternatives to recycle soil nutrients, supply organic matter and ultimately, restore eroded and overexploited soils - QS biochar can be relevant for environmental remediation applications in other agriculture sectors and regions of the country (e.g. Cd adsorption in cocoa crops) - Biochar can also be used as a feed supplement for cows and sheep’s husbandry or rainwater filtration - If surplus biochar and PCMs are marketed by local communities, can be a new source of familiar income - The alternative of using low-cost and complexity reactors as top-lit-up draft gasifiers or flame curtain kilns and retorts - Low-cost and complexity technologies could be replicated by other quinoa producers in the region, namely Perú and Bolivia which also face the consequences of soil over-exploitation - Creation of jobs in the rural sector: sales and operation of pyrolysis reactors (tech as a service), maintenance and management of pyrolysis facilities. Sale of surplus biochar - Carbon removal certificates that result from the application of biochar in soils could be claimed by farmers’ communities. The emergence of carbon sequestration standards tailored for small-scale farmers	- Environmental regulations: currently, there are no specific standards in Ecuador, nor in other producer countries as Peru and Bolivia to regulate the use and efficiency of pyrolysis reactors (e.g., flue gas emissions standards) - Indeed, there is no dedicated standard to regulate the biochar composition or its use in soils - The Ecuadorian constitution (Art 74) forbids the trade of carbon removal certificates generated from ecosystem services. Accordingly, monetization of carbon sequestration services using soil as carbon sinks is uncertain - High-cost international certification for farmers as providers of carbon sequestration services - Inadequate use of low-cost and low-complexity reactors to convert agro residues into biochar may result in low-quality products and dangerous gaseous emissions

The alternative to deploy a decentralized operation for the conversion of agro residues into biochar and PCMs, similar to the portable threshing service currently used, may not be an option because the pilot-scale auger-type pyrolysis reactor requires an electricity supply to power its electric devices and the automation system. According to our participant observations, the threshing machines use gasoline to produce mechanical work because electricity supply is not available on every farm. Thus, the implementation of a portable auger-type pyrolysis reactor to deliver pyrolysis as a farm delivery service may not be entirely feasible. In this context, Table 6 points as an opportunity the study, adaptation, and later implementation of low-complexity technologies for the production biochar, for instance, top lift updraft gasifiers TLUD´s or flame curtain retort kilns (Obi et al., 2016; Pandit et al., 2017). These types of low-cost reactors may allow the use of the agro residues on each farm, avoiding collection, transporting, and even milling operations. Nonetheless, the quality of the biochar produced in low-cost reactors must be carefully analyzed to guarantee its safe application for soil amendment. In this regard, the results presented in Section 3.4 concerning the properties and composition of the biochar and PCMs made from QS and QH are a major quality reference.

Moreover, a set of studies made in the same reactor used in this work and using operating conditions similar to that disclosed in Section 3.3 states that the biochar made from QS can be used to prevent cadmium absorption in aqueous solutions, reducing up to 71% of the bioavailable cadmium in acidic soils used to grow cocoa (López et al., 2020, 2022). Hence, the biochar made from quinoa agro residues could be relevant not only to prevent soil erosion at the local level but for a wide range of environmental remediation applications. Accordingly, Table 6 claims that the conversion of QS into biochar may generate new sources of income in rural areas, for instance, those derived from the marketing of surplus biochar for use in other sectors such as animal husbandry, water filtration or environmental remediation (Man et al., 2020).

During the participant observations, the farmers revealed that some farms have in place infrastructures for the elaboration of bio inputs such as compost, bokashi (organic fertilizer made by fermentation), and biols (liquid fertilizer made from anaerobic digestion of manure). Hence, the implementation of low-cost and complexity technologies for biochar production in the farms can be an opportunity to complement these infrastructures and potentially improve the bio inputs used in the farms. Our SWOT analysis also shows that the implementation of valorization technologies to convert agro residues generated locally into biochar or PCMs can be an opportunity to create jobs, for instance, those required to provide, manage, operate (tech as a service), repair, and maintain these pyrolysis reactors.

It is worth to highlight that application of biochar in soils by Andean farmers can result in carbon removal certificates, that properly traced and traded, could be another source of income for farmers (Heredia Salgado et al., 2021). However, complex technical and bureaucratic processes are required to register a farmer as a provider of carbon sequestration services (Bier et al., 2020; Schmidt et al., 2020). Currently, Ecuador does not have a technical standard to regulate the use of biochar in soils. In addition, there is an ongoing discussion regarding the interdict established by the Ecuadorian constitution (art 74), which prevents privates from appropriating and trading with services derived from ecosystems, for example, the carbon removal certificates, or carbon credits linked with carbon sequestration in soils, including those considering forestation and afforestation. Perhaps, the alternatives of carbon sequestration that are not linked with ecosystem services, for instance, the use of biochar as an additive in cement (sequestration in gray infrastructures, buildings, dams, *etc.*) may be out of the constitution interdict. Nonetheless, great uncertainty remains as to whether the implementation of pyrolysis facilities for biochar production can turn farmers into providers of carbon sequestration services. In this regard, the lack of local standards and regulations constitute a threat that is currently preventing the mobilization of funds for the implementation of alternatives for the conversion of agro residues into biochar and PCM´s.

## 4 Conclusion

The studies that support the use of biochar in quinoa and lupin crops and that validate its effect on the restoration of degraded ecosystems have been performed using biochars produced from agro residues not necessarily available in the Andean highlands, for example, peanut hull residues. Biochar could become an alternative to improve soil management practices and material to restore the overexploited ecosystems during the quinoa and lupin boom, to the extent that the agro residues available in the Andean highlands may be used as the feedstock of the pyrolysis process. Our study shows that the agro residues generated after the threshing processes of quinoa, namely QS and QH can be transformed into materials for soil amendment using a pilot-scale auger-type pyrolysis reactor.

Following the European guidelines for the sustainable production of biochar, the solid carbonaceous material produced from QS pyrolysis can be categorized as biochar while the carbonaceous material produced from QH pyrolysis can be categorized as pyrogenic carbonaceous material (PCM). The agro residues generated during lupin threshing, namely lupin stems and lupin seedcases were not properly pyrolyzed. Our study further shows that the concentration of CO in the flue gas observed during the pyrolysis of QS and QH was 1,024.4 and 559 mg/Nm^3^, respectively. The experiments were performed in the Andes highlands at an altitude of 2,634 m.a.s.l., that is, the oxygen concentration in the air decreased from 21 to 16% due to the decrease in atmospheric pressure. Despite the low oxygen concentration in the air, we observed that the differences in the composition of the flue gas in these experiments were influenced by the individual free fall density of the agro residues particles. Accordingly, a steady flow of QH particles in the reactor hopper and along the pyrolysis chamber resulted in a CO concentration of 559 mg/Nm^3^ which is near the European eco-design standard of 500 mg/Nm^3^.

From the participant observations and subsequent SWOT analysis, we claim that the implementation of an auger-type pyrolysis reactor may not be feasible in practice whether the operation model considers a centralized or decentralized valorization of agro residues. A centralized operation using an auger-type pyrolysis reactor will result in high collection, transportation and operating costs. Likewise, a decentralized operation will also be problematic because the electricity required to power the automation and electric devices as controllers, motors and blowers is not usually available in remote farms. Accordingly, we suggest the study and adaptation of low-cost and complexity reactors as an alternative for the decentralized pyrolysis of quinoa and lupin agro residues, namely, the top-lit updraft gasifiers (TLUD) and flame curtain kilns. Unlike low-cost and complexity reactors, the auger-type pyrolysis reactors allow a precise control of operating conditions such as pyrolysis temperature and residence time, also resulting in lower flue gas emissions. Nonetheless, if the quality of biochar is not compromised, the low-cost and low complexity reactors may constitute alternatives for the conversion of agro residues on each farm avoiding collection, transportation and even milling expenditures. In this regard, the properties of the biochar and PCM’s along with the operating conditions disclosed in this study will serve for guidance and reference. Finally, the lack of local standards that regulate the production and use of biochar could make it difficult for local stakeholders as farmers’ cooperatives, NGOs, the government, or quinoa/lupin exporting companies to advance with implementation models of TLUD’s or flame curtain kilns at larger scales.

## Data Availability

The raw data supporting the conclusions of this article will be made available by the authors, without undue reservation.
